# Construction of the coexpression network involved in the pathogenesis of thyroid eye disease via bioinformatics analysis

**DOI:** 10.1186/s40246-022-00412-0

**Published:** 2022-09-08

**Authors:** Jinxing Hu, Shan Zhou, Weiying Guo

**Affiliations:** 1 Department of Endocrinology, HwaMei Hospital, University of Chinese Academy of Sciences, 41 Northwest Street Zhejiang Province, Ningbo, 315010 China; 2 Ningbo Institute of Life and Health Industry, University of Chinese Academy of Sciences, Ningbo, 315010 China

**Keywords:** Gene coexpression networks, Thyroid eye disease, Autoimmune inflammatory disease, Differentially expressed genes, Protein–protein interaction

## Abstract

**Background:**

Thyroid eye disease (TED) is the most common orbital pathology that occurs in up to 50% of patients with Graves’ disease. Herein, we aimed at discovering the possible hub genes and pathways involved in TED based on bioinformatical approaches.

**Results:**

The GSE105149 and GSE58331 datasets were downloaded from the Gene Expression Omnibus (GEO) database and merged for identifying TED-associated modules by weighted gene coexpression network analysis (WGCNA) and local maximal quasi-clique merger (lmQCM) analysis. EdgeR was run to screen differentially expressed genes (DEGs). Transcription factor (TF), microRNA (miR) and drug prediction analyses were performed using ToppGene suite. Function enrichment analysis was used to investigate the biological function of genes. Protein–protein interaction (PPI) analysis was performed based on the intersection between the list of genes obtained by WGCNA, lmQCM and DEGs, and hub genes were identified using the MCODE plugin. Based on the overlap of 497 genes retrieved from the different approaches, a robust TED coexpression network was constructed and 11 genes (ATP6V1A, PTGES3, PSMD12, PSMA4, METAP2, DNAJA1, PSMA1, UBQLN1, CCT2, VBP1 and NAA50) were identified as hub genes. Key TFs regulating genes in the TED-associated coexpression network, including NFRKB, ZNF711, ZNF407 and MORC2, and miRs including hsa-miR-144, hsa-miR-3662, hsa-miR-12136 and hsa-miR-3646, were identified. Genes in the coexpression network were enriched in the biological processes including proteasomal protein catabolic process and proteasome-mediated ubiquitin-dependent protein catabolic process and the pathways of endocytosis and ubiquitin-mediated proteolysis. Drugs perturbing genes in the coexpression network were also predicted and included enzyme inhibitors, chlorodiphenyl and finasteride.

**Conclusions:**

For the first time, TED-associated coexpression network was constructed and key genes and their functions, as well as TFs, miRs and drugs, were predicted. The results of the present work may be relevant in the treatment and diagnosis of TED and may boost molecular studies regarding TED.

**Supplementary Information:**

The online version contains supplementary material available at 10.1186/s40246-022-00412-0.

## Background

Thyroid eye disease (TED), also known as thyroid-associated ophthalmopathy (TAO), Graves’ ophthalmopathy or thyroid orbitopathy, is one of the common autoimmune diseases behind the eye. This disease is commonly seen with Graves’ disease and Hashimoto’s thyroiditis, but can also occur in healthy people and patients with hyperthyroidism [1]. TED is caused by systemic autoimmune attacks of the orbit and other targeted tissues, such as thyroid, skin, pretibial soft tissues [2], periorbital connective tissue, extraocular muscles and orbital fat tissue. Its symptoms include mild eye irritation, severe disfigure and even permanent blindness [3]. Although the majority of TED cases exhibit only mild eye disease, 3–5% of cases exhibit vision loss mainly due to exposure corneal ulceration or compressive optic neuropathy [4]. TED is the result of a combination of genetic and environmental factors. Many of these factors, including race, gender, age, smoking history, thyroid hormone, radioactive stimulating hormone (RAI) therapy and thyroid-stimulating hormone receptor antibodies, have been confirmed by numerous studies [5]. A previous study suggested that the immune response is an important factor involved in TED [6]. However, the pathologic mechanism of TED is still poorly understood. Understanding the pathological mechanism of TED will lead to the discovery of targeted molecular approaches for TED and provide strong evidence for the future treatment of TED. Therefore, it is necessary to scrutinize the molecular mechanism of TED, especially the key genes and pathways related to TED.

Up to date, the transcriptome data of TED are very limited. In addition to data limitation, the methods used for identifying genes with biological relevance in TED are also limited to differential expression analysis-based approaches. Although studies reported by Rosenbaum et al. [6], Zhu and Yang [7] and Lee et al. [8] identified some genes related to TED, the unicity of their approach may not help capture some highly TED-related genes. Thus, integrating multiple approaches may help identify the credible key genes associated with TED.

Gene coexpression networks (GCNs) are key tools for identifying molecular mechanism of diseases [9]. Their main steps include calculating coexpression and selecting important thresholds to filter networks. GCNs can be used to screen candidate biomarkers and therapeutic targets [10, 11]. Although this approach cannot provide a causal relationship, the coexpression network can find regulatory genes in many phenotypes [12]. The weighted gene coexpression network analysis (WGCNA) is a systematic biological method used for detecting coexpressed genes that cannot be detected by differential analysis and has good applications in microarray data or deep sequencing. The problem of multiple tests inherent in microarray data analysis is alleviated in WGCNA [10] which studies biological networks based on pairwise correlations between variables, which is suitable for most high-dimensional data sets. Although principal component analysis (PCA) can also handle high-dimensional data, this method provides very little information [13]. Thus, WGCNA is usually performed to identify modules containing a high correlation with disease characteristics. Generally, WGCNA is a widely implemented in the identification of gene modules involved in diverse diseases. For example, in the study of lupus arthritis (LA), researchers identified highly coexpressed gene modules associated with LA through WGCNA analysis, revealed immune/inflammatory cells dominated by myeloid phenotype and identified cell composition and physiological pathways that are relevant in LA [14]. Researchers have also identified key modules in the psoriatic arthritis (PsA) dataset GSE61281 through WGCNA; through GO and KEGG analysis of key modules and other analysis tools, they determined that RHOH/TRAF1 has great effects on the pathogenesis of PsA [15]. WGCNA has been also widely applied in autoimmune diseases [16, 17] and cancer [18, 19] research. WGCNA was implemented by Li and colleagues in the research of rheumatoid arthritis and allowed the discovery of 4 key modules associated with RA, revealing the biological processes and pathways related to immunity and infection involved in RA [20]. The WGCNA also bridges the gap between individual genes and systemic oncology [21]. WGCNA is not only used for identifying coexpressed mRNAs, but also coexpressed microRNA and lncRNAs. For example, Giulietti et al. [22] discovered the expression of LINC00675 and LINC01133 lncRNAs as well as the occurrence and development of pancreatic cancer by using the coexpression network. Zhou et al. [23] found that hsa-mir-125b-5p, hsa-mir-145-5p, hsa-let-7c-5p, hsa-mir-218-5p and hsa-mir-125b-2-3p were the prognostic and pathological hub miRNAs involved in colon cancer by using WGCNA. However, WGCNA has not been applied for identifying coexpression networks involved in TED. In addition to the widely used WGCNA, there are also GCNs such as mutual rank, highest reciprocal ranks (HRR) and lmQCM [24, 25]. lmQCM is a new network mining method that can be used to identify densely connected modules [26]. The main feature of this method is the use of the local maximum pass to initialize the search, avoid excessive overlap between modules and improve the efficiency of calculation. In addition, this method includes the weight normalization process and can find modules with more balanced sizes. lmQCM has been used in cancer and neurodegenerative syndrome [25, 27]. In a latest report, lmQCM has been used to analyze rhabdomyosarcoma subtypes [28] and 41 coexpression modules were found, of which 17/41 showed obvious up-regulation or down-regulation in the fusion state. However, lmQCM has not been applied to screen coexpression modules associated with TED. Thus, applying WGCNA and lmQCM to TED-related transcriptome data is promising in discovering new key genes and pathways involved in TED.

Therefore, in our present study, the WGCNA and lmQCM algorithms were employed to generate gene coexpression modules associated with TED. The key genes obtained from different approaches were used for TED coexpression network construction, followed by the identification of TFs, hub genes and miRs that may participate in TED pathogenesis. Potential therapeutics were also predicted based on coexpression network genes. The present work may promote the understanding of the pathological mechanism of TED.

## Results

### Analysis and functional enrichment analysis of the DEGs

**Fig. 1:**
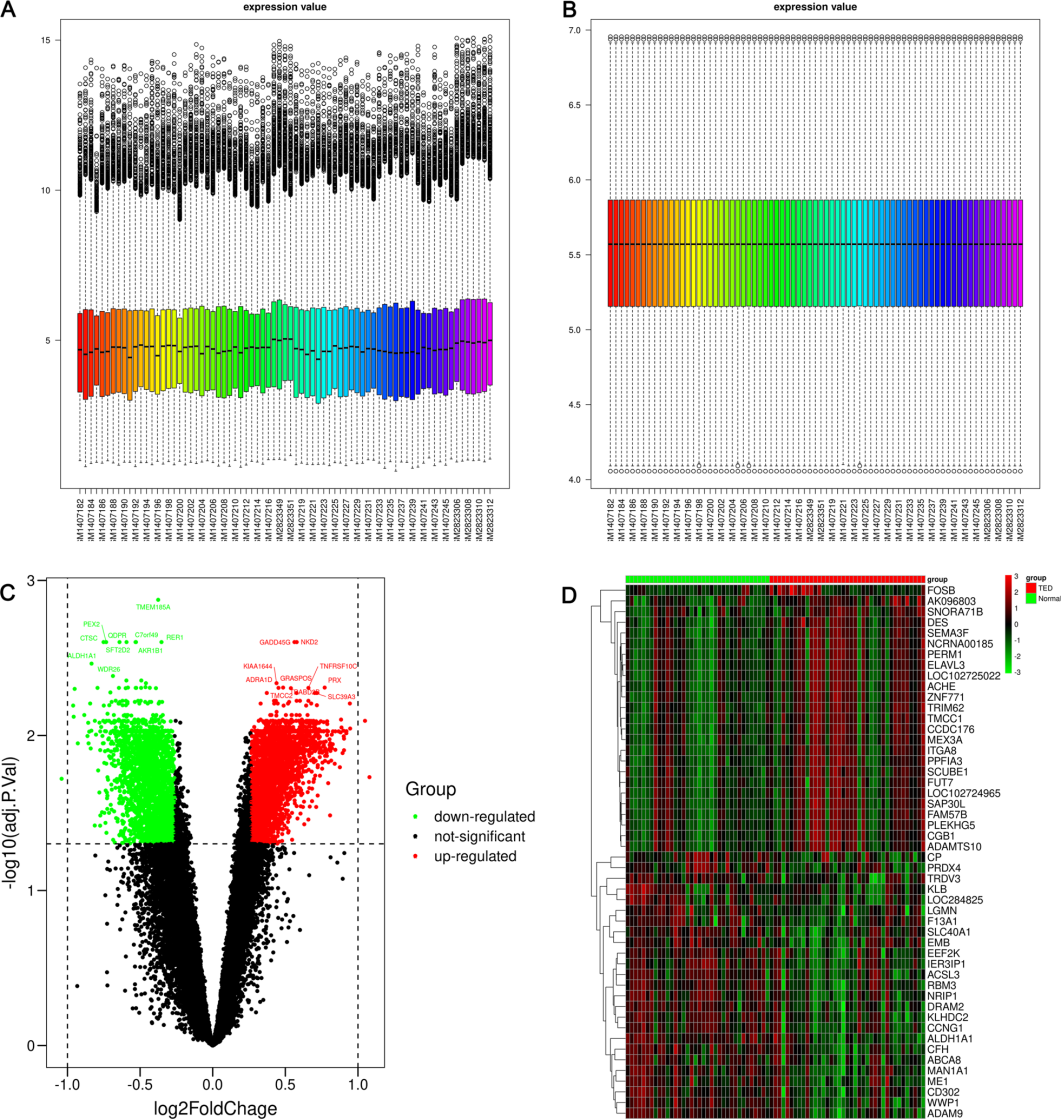
Data preprocessing and differential expression gene analysis. Boxplot of the merged matrix of transcriptome data **A** before and **B** after batch effect removal and normalization. **C** Volcano plot of DEGs. Top 10 DEGs (sorted by adj. *p* value) in the up-regulated and down-regulated groups. **D** Heatmap of DEGs. Top 10 DEGs (sorted by |log2FC|) in the up-regulated and down-regulated groups

Through differential expression analysis, we obtained 4,999 DEGs (2767 up-regulated, 2232 down-regulated) between TED and normal samples. The volcano plot and heatmap of DEGs are shown in Fig. 1C, D, respectively. As shown in Additional file 1: Table S1, the DEGs were enriched in the biological process (BP) terms of Golgi vesicle transport, positive regulation of establishment of protein localization, positive regulation of cellular protein localization and endoplasmic reticulum to Golgi vesicle. The predominant molecular function (MF) terms were endopeptidase activity, ubiquitin-like protein ligase binding and ubiquitin protein ligase binding. For the cellular component, the most enriched terms were mitochondrial matrix, nuclear speck and transport vesicle. The KEGG pathways of the DEGs were enriched in MAPK signaling pathway, endocytosis and protein processing in endoplasmic reticulum.

### Construction of weighted gene coexpression networks and functional enrichment analysis

**Fig. 2:**
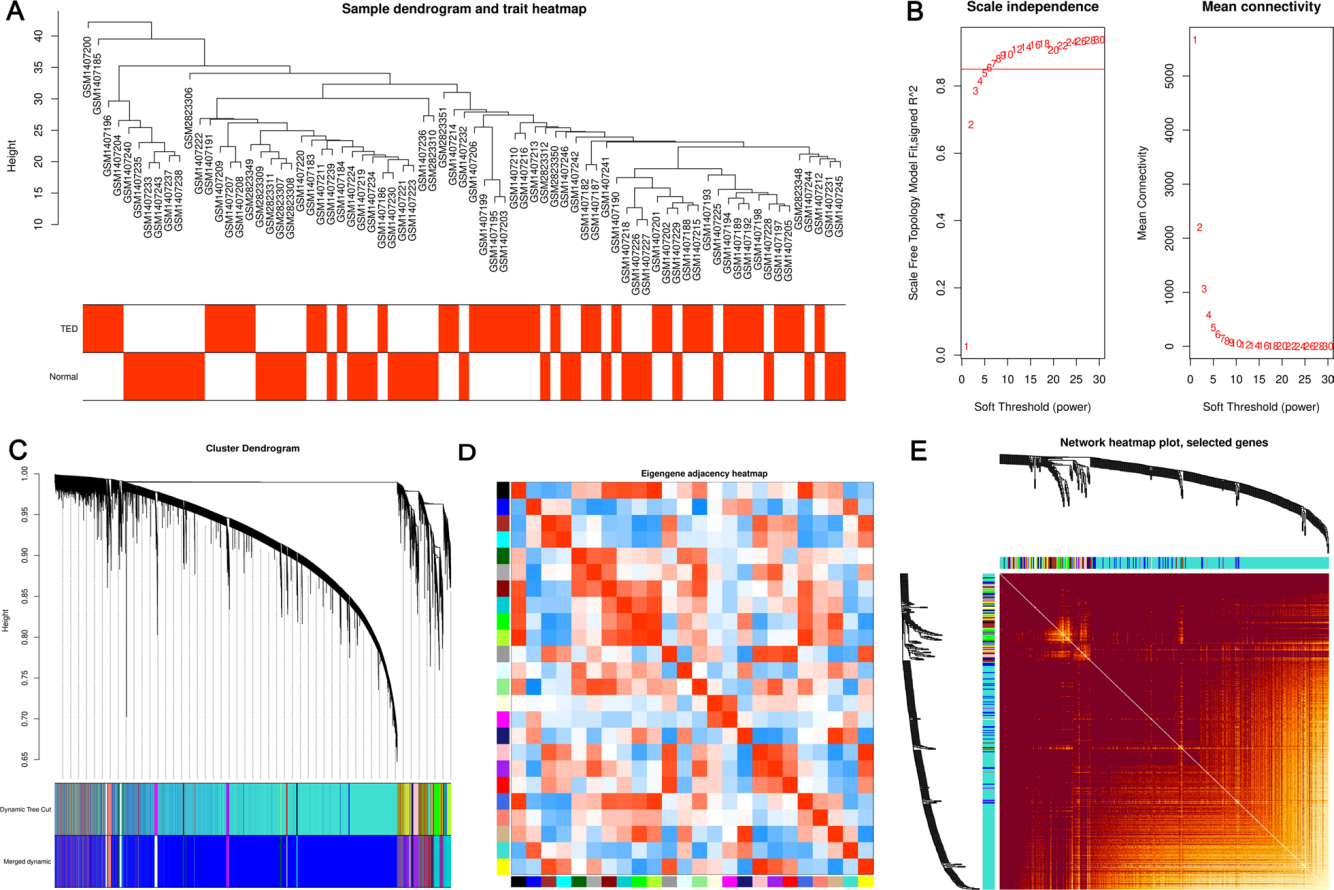
Construction of WGCNA networks. **A** Sample dendrogram and trait heatmap. The two traits are TED and normal. **B** Scale independence and mean connectivity of various soft-thresholding values (*β*). **C** Gene dendrogram and modules color. **D** Eigengene adjacency heatmap. **E** The heatmap of the 400 genes in the coexpression network

**Fig. 3:**
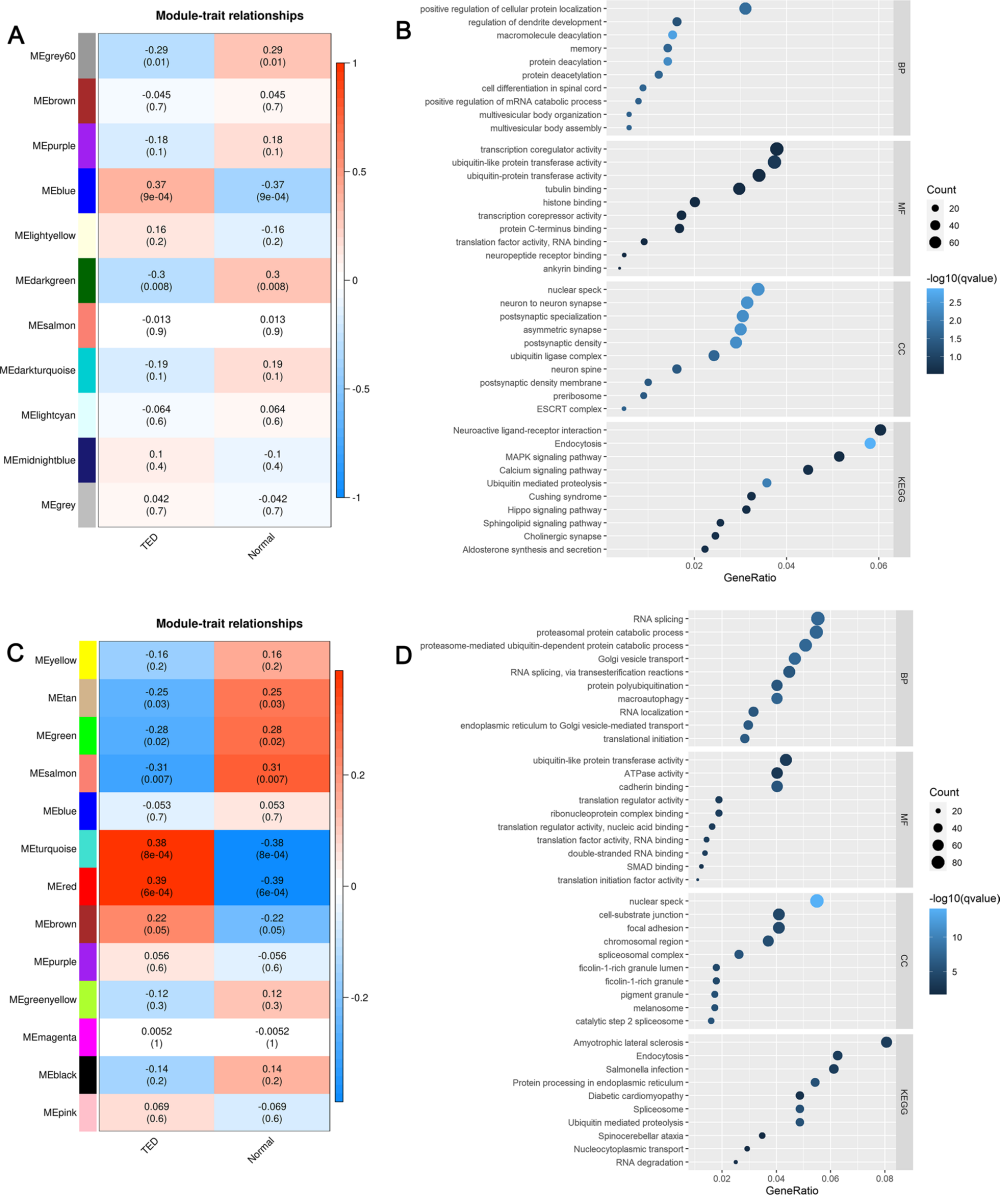
WGCNA and lmQCM modules and functional analysis of key modules. **A** Module–trait relationships in WGCNA modules. **B** Function enrichment analysis of the blue module most significantly correlated with TED as identified by WGCNA. **C** Module–trait relationships in lmQCM modules. **D** Function enrichment analysis of the red module most significantly correlated with TED as identified by lmQCM

The sample dendrogram and trait heatmap of samples in the TED matrix are shown in Fig. 2A. In WGCNA analysis, TED coexpression network was built when the power value equaled 6 (Fig. 2B). The cluster dendrogram showing the dynamic cut and merging of merged modules is depicted in Fig. 2C. The heatmap of eigengene adjacency is reported in Fig. 2D, while the network heatmap of selected genes is reported in Fig. 2E. Similarly, another TED coexpression network was also constructed by lmQCM analysis. Finally, we got 11 WGCNA modules (Fig. 3A) and 13 lmQCM modules (Fig. 3C). Based on the correlation between MEs and TED trait in the modules among WGCNA modules or lmQCM modules, we selected the modules most highly correlated to TED as TED-specific modules. The blue module (correlation = 0.37, *P* value = 0.0009) containing 17,008 genes was the module most correlated with TED among modules identified by WGCNA and was selected as the key TED-associated module for further analysis (Fig. 3A). The functional enrichment analysis of genes in the blue (Fig. 3B, Additional file 2: Table S2) indicated that these genes were enriched in the biological processes (BP) of positive regulation of cellular protein localization, regulation of dendrite development, macromolecule deacylation, memory, protein deacylation, protein deacetylation, cell differentiation in spinal cord and positive regulation of mRNA catabolic process. In the category of molecular function (MF), “transcription coregulator activity” and “ubiquitin-like protein transferase activity” were the most significantly enriched terms, whereas in the category of cellular component (CC), “nuclear speck,” “neuron to neuron synapse” and “postsynaptic specialization” were the most enriched terms (Fig. 3B, Additional file 2: Table S2). The pathways of the genes in the blue module were neuroactive ligand–receptor interaction, endocytosis, MAPK signaling pathway, ubiquitin-mediated proteolysis, Cushing syndrome, hippo signaling pathway, sphingolipid signaling pathway, cholinergic synapse and aldosterone synthesis and secretion (Fig. 3B, Additional file 2: Table S2). The detailed information can be found in Additional file 2: Table S2.

Among the lmQCM modules (Fig. 3C), the module most correlated with TED was the red module (correlation = 0.39, *P* value = 0.0006) which contained 1873 genes. We found that genes in the red module obtained from lmQCM analysis (Fig. 3D, Additional file 3: Table S3) were mostly enriched in the BP terms of RNA splicing, proteasomal protein catabolic process, proteasomal-mediated ubiquitin-dependent protein catabolic process and Golgi vesicle. The most enriched MF terms of genes in the red module were “ubiquitin-like protein transferase activity,” “ATPase activity,” “cadherin binding” and “translation regulator activity” while the most enriched CC terms were “nuclear speck,” “cell-substrate junction,” “focal adhesion,” “chromosomal region” and “spliceosomal complex” (Fig. 3D, Additional file 3: Table S3). For the KEGG pathway analysis, amyotrophic lateral sclerosis, endocytosis, Salmonella infection, protein processing in endoplasmic reticulum, diabetic cardiomyopathy, ubiquitin-mediated proteolysis and spinocerebellar ataxia were the most enriched pathways (Fig. 3D, Additional file 3: Table S3). The more detailed information about the modules identified by lmQCM is summarized in Additional file 3: Table S3.

### Construction of a robust TED coexpression network and identification of hub genes

**Fig. 4:**
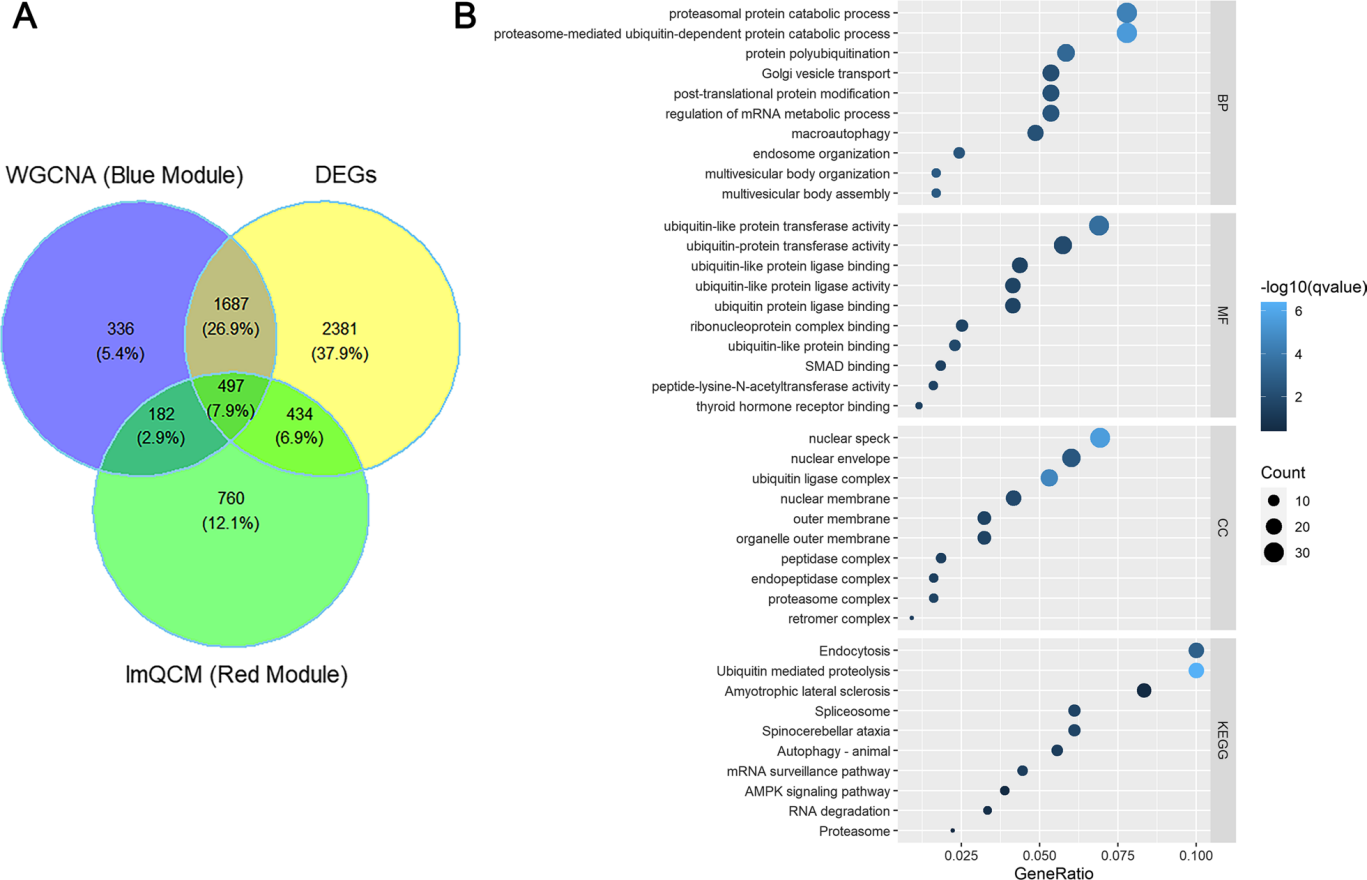
The overlap of DEGs and genes in key TED-associated modules identified by WGCNA and lmQCM. **A** The Venn plot of the DEGs and genes in key TED-associated modules identified by WGCNA and lmQCM. **B** The function enrichment analysis of the overlap of DEGs and genes in key TED-associated modules identified by WGCNA and lmQCM

**Fig. 5:**
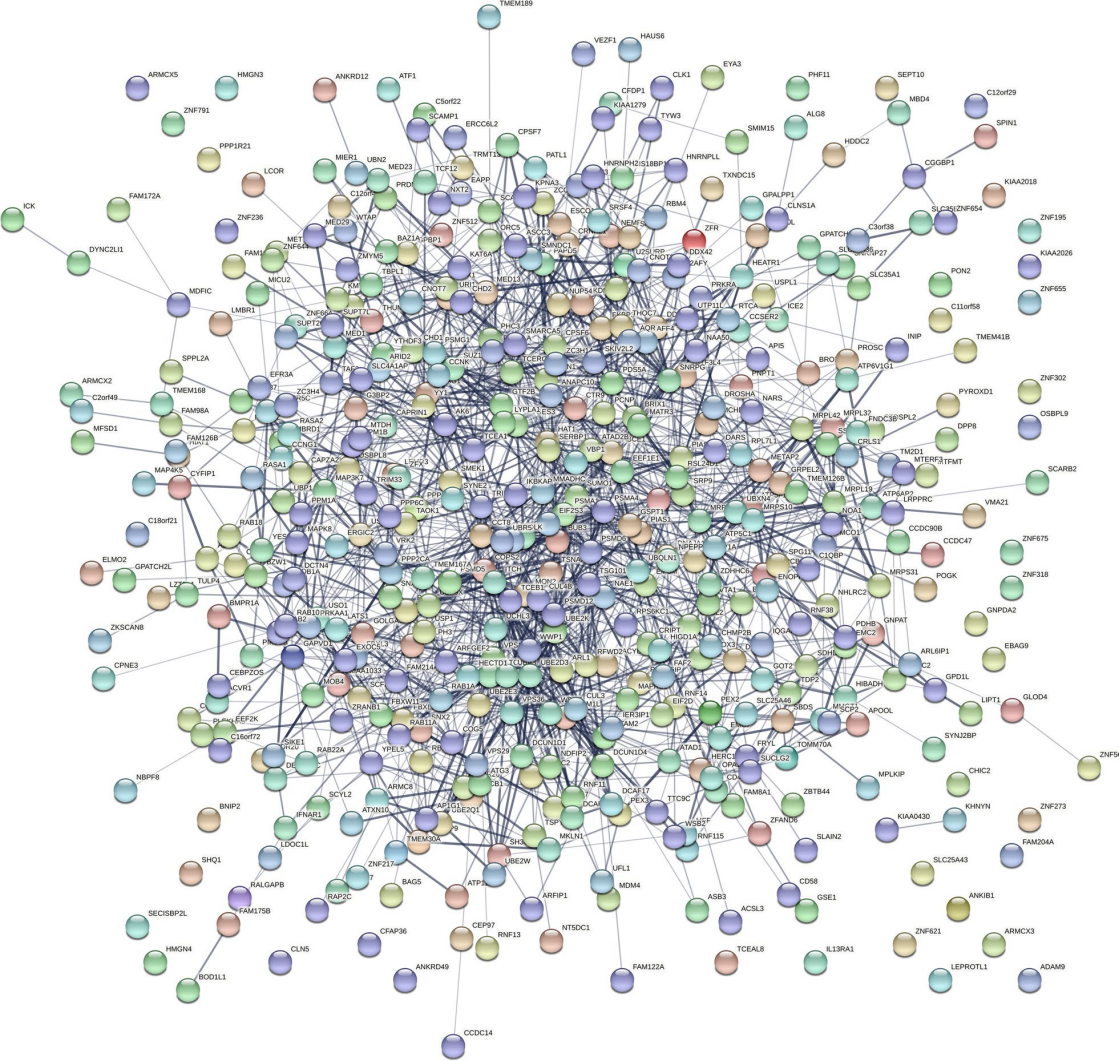
Construction of a robust TED coexpression network based on the intersection genes. The 497 coexpression genes obtained from the intersection of the overlap of DEGs and genes in key TED-associated modules identified by WGCNA and lmQCM were imported in stringdb for protein–protein interaction (PPI) network, and the PPI network was downloaded

**Fig. 6:**
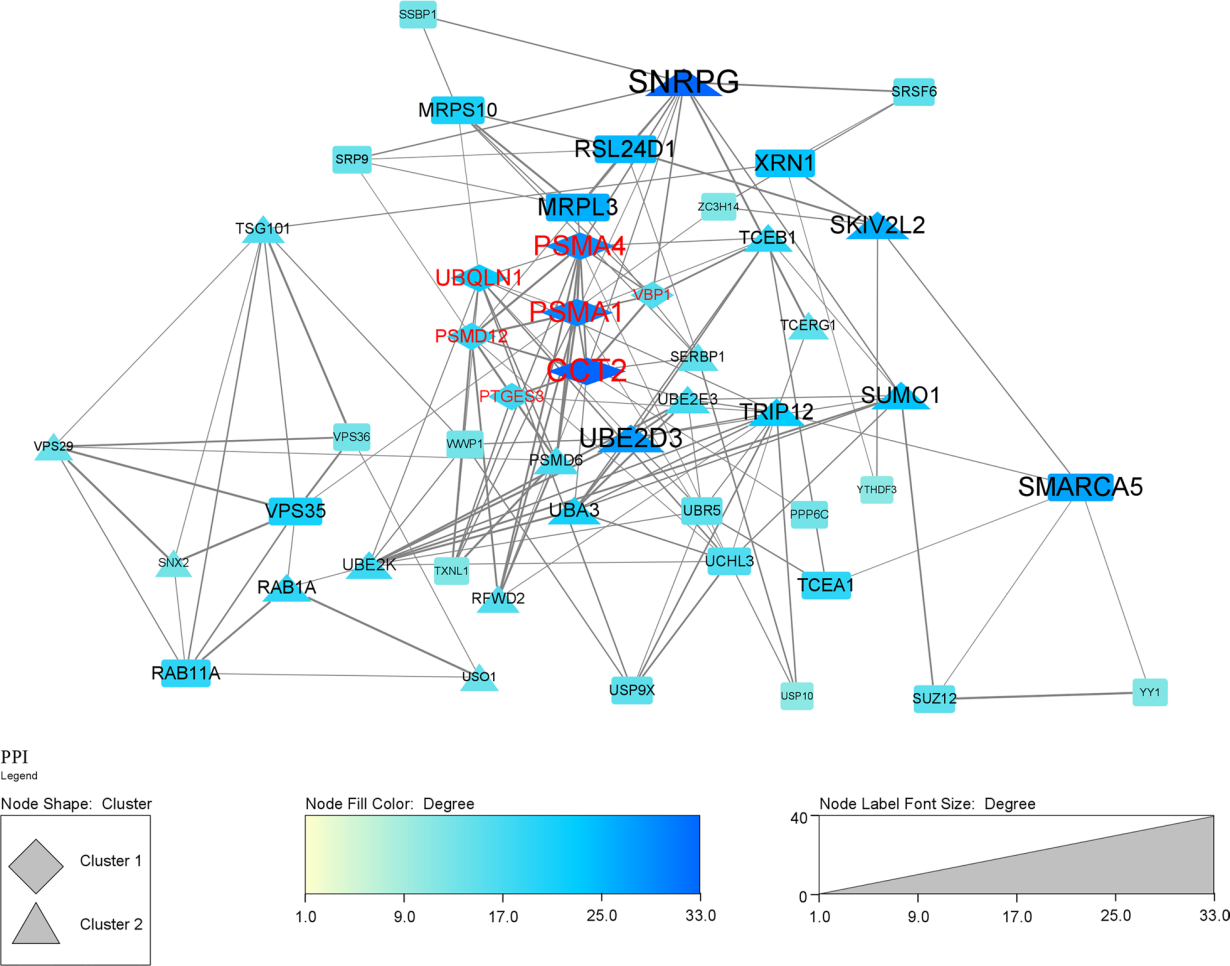
Visualization of the coexpression network in Cytoscape and identification of clusters and hub genes. The PPI network of the TED coexpression genes was visualized in Cytoscape. Clusters were identified by MCODE, and genes with node degree higher than ten were visualized. Node labels in red indicated hub genes with node degree higher than ten

A total of 497 overlap genes were obtained by intersecting the DEGs and genes in the blue (WGCNA) and red (lmQCM) modules associated with TED (Fig. 4A). The enrichment analysis of the TED-specific genes (Fig. 4B, Additional file 4: Table S4) demonstrated that the genes were enriched in “proteasomal protein catabolic process,” proteasome-mediated ubiquitin-dependent protein catabolic process, protein polyubiquitination, Golgi vesicle transport, post-translational protein modification, regulation of mRNA metabolic process, macroautophagy and endosome organization in the category of BP. In the MF category, ubiquitin-like transferase activity, ubiquitin transferase activity, ubiquitin-like protein ligase binding and ubiquitin-like protein ligase activity were the most enriched terms (Fig. 4B, Additional file 4: Table S4). In the CC category, nuclear speck, nuclear envelope and ubiquitin ligase complex were the most enriched terms (Fig. 4B, Additional file 4: Table S4). For KEGG pathways, endocytosis, ubiquitin-mediated proteolysis, amyotrophic lateral sclerosis, spliceosome, spinocerebellar ataxia, autophagy in animal, mRNA surveillance pathway, AMPK signaling pathway, RNA degradation and proteasome were the most enriched pathways of the 497 overlap genes (Fig. 4B, Additional file 4: Table S4). Then, the 497 overlap genes were used as input for PPI analysis. PPI network was constructed in STRING to explore the interactions between genes involved in TED. The PPI network generated from the overlap genes included 443 nodes and 1650 edges, and the average number of neighborhoods was 7.478 (Fig. 5). For clearer visualization, we kept nodes with degree greater than ten (Fig. 6). MCODE analysis allowed the identification of 2 predominant clusters of hub genes. The 11 hub genes in cluster 1 with the highest score were ATP6V1A, PTGES3, PSMD12, PSMA4, METAP2, DNAJA1, PSMA1, UBQLN1, CCT2, VBP1 and NAA50, among which PTGES3, PSMD12, PSMA4, PSMA1, UBQLN1, CCT2 and VBP1 had node degree higher than ten.

### Prediction of transcription factors (TFs), miRs and drugs targeting genes in the robust TED coexpression network

We uploaded TED-specific genes to ToppGene to investigate the potential TFs, miRs and drugs perturbing the biological function and pathways involved in TED pathogenesis. Detailed information of the TFs is summarized in Additional file 5: Table S5. Based on the overlap genes, we retrieved 221 TED-associated TFs at a *p* value cutoff of 0.01. As shown in Additional file 5: Table S5, NFRKB had the highest number (115 genes) of regulatory targets among genes in the coexpression network, followed by ZNF711 (104 genes), ZNF407 (97 genes), MORC2 (91 genes), NFE2L1 (89 genes), UBP1 (87 genes) and HOXA2 (86 genes). In total, 476 genes in the TED-associated coexpression network were targeted by the predicted TFs. To further investigate the regulation of genes in the TED coexpression network, ToppGene was used for the prediction of 4581 miRs targeting these genes from different databases. As shown in Additional file 5: Table S5, we found that hsa-miR-144 had the highest number of targets (134 genes) among genes in the coexpression network, followed by hsa-miR-3662 (130 target genes), hsa-miR-12136 (126 target genes), hsa-miR-3646 (122 target genes), hsa-miR-607 (121 target genes), hsa-miR-548x-3p (120 target genes), hsa-miR-548aj-3p (120 target genes) and hsa-miR-411* (116 target genes). Moreover, for therapeutic purpose, we predicted the drugs targeting the genes in the TED-associated coexpression network. As shown in Additional file 5: Table S5, 138 drugs were predicted to significantly target genes in the coexpression networks. Among these drugs, enzyme inhibitors had the highest number of target genes (112 genes), followed by the chlorodiphenyl (103 target genes), finasteride (90 target genes), nefazodone (89 target genes) and sodium arsenate (85 target genes).

## Discussion

Thyroid eye disease (TED) is one of the most common autoimmune inflammatory diseases [29]. In about 3–5% of patients, TED is accompanied by loss of vision and compressive optic neuropathy [4]. Exploring disease-related genes on the molecular level can contribute to the development of drugs for TED treatment. In this study, we used the differential expression analysis, WGCNA and lmQCM approaches to identify TED-associated modules and constructed a solid coexpression network for TED. Based on the overlap of important genes retrieved from the different approaches, 11 genes (ATP6V1A, PTGES3, PSMD12, PSMA4, METAP2, DNAJA1, PSMA1, UBQLN1, CCT2, VBP1 and NAA50) were identified as hub genes in the PPI network of the overlap coexpression genes. We also found that NFRKB, ZNF711, ZNF407 and MORC2 were key candidate TFs possibly regulating genes in the TED-associated coexpression network. Similarly, the genes in the coexpression network were mostly regulated by predicted miRs such as hsa-miR-144, hsa-miR-3662, hsa-miR-12136 and hsa-miR-3646. Functional enrichment analysis indicated that genes in the coexpression network were enriched in the biological processes related to proteasomal protein catabolic process, proteasome-mediated ubiquitin-dependent protein catabolic process and protein polyubiquitination and the pathways of endocytosis, ubiquitin-mediated proteolysis and amyotrophic lateral sclerosis. Furthermore, drug prediction based on the coexpression network indicated that most of genes were targets for enzyme inhibitors, chlorodiphenyl, finasteride and nefazodone. The study is the first, to the best of our knowledge, to construct such a stringent coexpression network for TED and to explore its regulatory factors and potential targeting drugs. The present findings are important in the understanding of the pathogenesis of TED, its diagnostics and the development of drugs for this disease.

Previous studies have revealed the clinical features and some key genes associated with TED. For instance, Grusha Ia et al. [30] found that the expression of HBD-2 may cause corneal damage in patients. Previously, several researchers have also identified hub genes by using bioinformatics analysis. Lee et al. [8] identified that 328 DEGs were associated with active TED through RNA sequencing. Many of these were associated with inflammation, cytokine signaling, adipogenesis, IGF-1 signaling and glycosaminoglycan binding. Gopinath et al. [3] demonstrated that cardiac calsequestrin gene CASQ2 was markedly up-regulated (~ 2.2-fold) in patients with the orbital disease and/or healthy people by microarray analysis. Zhu and Yang [7] indicated that POMC, IL-2, GNG3, CXCR4, TLR4, CSF1R, LPAR3 and CXCL8 were hub genes among many DEGs. It was also found that 71 DEGs were associated with TED in the comparative Toxicogenomics Database. Rosenbaum et al. [6] extracted RNA from the orbit of 83 volunteers and found that TED had fewer inflammatory marker characteristics than other ophthalmologic diseases through PCA. In the present study, by combining multiple bioinformatics approaches, we were able to construct, for the first time, a robust regulatory coexpression network. The regulatory interaction genes identified here might shed light in the understanding of the pathogenesis of TED.

There are various tools for weighted gene coexpression network mining, for example, quasi-clique merger (QCM), lmQCM, Markov clustering (ML) and WGCNA. Except for WGCNA, lmQCM is suitable for identifying small yet densely connected gene coexpression networks. A detailed description of each approach is provided in “Introduction” and “Methods” sections. Here, we performed lmQCM and WGCNA network and found that 11 and 13 modules were identified by WGCNA and lmQCM, respectively. The correlation between TED and both lmQCM modules and WGCNA modules was similar, ranging from − 0.31 to 0.39. However, we obtained a small number of genes in TED-specific modules through lmQCM compared with WGCNA. Moreover, 1488 genes of TED-specific modules were found in both WGCNA modules and lmQCM modules, suggesting that the genes obtained by both methods were the most credibly related to TED and that both methods are highly efficient in module discovery. Pathway enrichment analysis results showed that the pathways regulated by the genes in the TED-specific modules identified by lmQCM and WGCNA showed overlap in functional terms, indicating that both WGCNA and lmQCM were able to extract important genes involved in TED.

In eukaryotes, the binding of transcription factors and histone modification can accurately regulate gene expression. TFs can control the transcription rate of genetic information from DNA to messenger RNA by binding to specific DNA sequences. A number of studies have reported that transcription factors can participate in the activation and shutdown of gene functions. In order to explore the transcription factors regulating the expression of genes involved in TED, we used ToppGene to predict the TFs that regulate the expression of TED-specific genes. We found a total of 221 TFs that may be related to the expression of TED-associated coexpression genes. Among them, NFRKB, ZNF711, ZNF407 and MORC2 were the most prevalent, but these TFs and many of other identified TFs have not been reported in TED before. Thus, we can conclude that we identified new TED-related TFs which might be important in the research on the pathogenesis of TED. More relevant evidence needs to be provided in future research.

Ubiquitination is believed to be involved in the regulation of almost all life activities, including cell cycle, proliferation, apoptosis, gene expression, transcription regulation, inflammatory immunity, etc. Several studies have found that ubiquitination has an important regulatory role in autoimmune diseases [31–33]. Ubiquitination controls the development, activation and differentiation of T cells and maintains an effective adaptive immune response to pathogens and immune tolerance to self-tissues. Autoimmune diseases are not only directly affected by ubiquitination, but may also be regulated by ubiquitination-related enzymes. Wang and his colleagues found that mutations in the ubiquitin-conjugating enzyme E2L3 (UBE2L3) gene may be related to the high risk of Hashimoto’s thyroiditis (HT) in Han Chinese, indicating that ubiquitination may be involved in thyroid-related autoimmune diseases [34]. TED is an autoimmune disease, and its occurrence and development may be closely related to ubiquitination. In addition, a previous study reported that small ubiquitin-like modifier 4 (SUMO4) is a common autoimmune disease susceptibility gene in the Japanese population [35]. We found that the genes in the regulatory coexpression network were enriched in the biological processes related to proteasomal protein catabolic process, proteasome-mediated ubiquitin-dependent protein catabolic process and protein polyubiquitination and the pathway of ubiquitin-mediated proteolysis, indicating that ubiquitination plays a significant role in the pathogenesis of TED. The ubiquitin–proteasome pathway is responsible for the degradation of most intracellular proteins in eukaryotic cells [36]. Proteasome dysfunction is related to autoimmune diseases [37], neurodegeneration [38], heart [39] and other diseases. We found that genes in the regulatory network were also enriched in the proteasome pathway and the processes of proteasomal protein catabolic process, and proteasome-mediated ubiquitin-dependent protein catabolic process, which can explain a previous finding that the immune proteasome LMP2 can be used as a new target for the treatment of autoimmune hypothyroidism [40]. A previous study also demonstrated that TNF-α can promote mTOR-dependent proteasome-mediated PDCD4 degradation in orbital fibroblasts and that rapamycin can enhance TNF-α induction and IL6 secretion by inhibiting PDCD4 degradation in orbital fibroblasts [41]. Thus, the coexpression network constructed in this study may regulate the pathogenesis of TED via the ubiquitin–proteasome pathway (UPP). Although we only discussed some of the pathways, other pathways also deserve a more in-depth study.

In order to identify the interaction between proteins associated with the pathogenesis of TED and the related hub genes, we performed PPI analysis. We found 11 hub genes (ATP6V1A, PTGES3, PSMD12, PSMA4, METAP2, DNAJA1, PSMA1, UBQLN1, CCT2, VBP1 and NAA50) in the PPI network. Several studies reported that the PSMD12, PSMD14, PSMD5, PSMD6 were associated with the pathway of the proteasome [42–45]. PSMA1, PSMA4 and PSMB7 are the three subunits of the 20S proteasome [46]. The regulatory effects of PSMA1 and PSMD12 in the liver of thyroid hormone were previously reported [47], which is consistent with the down-regulation of PSMA1 and PSMD12 in TED as observed in the present work. The 11 hub genes may regulate TED by regulating the pathway of UPP, which is consistent with the findings in the functional enrichment analysis. At present, there is still little about the role of these hub genes in the regulation of thyroid diseases. Although we have discussed the regulatory function of PSMA1, PSMD12 in the thyroid, there is no report indicating that the 11 hub genes are directly related to the occurrence and progression of TED, and thus, more studies are needed to validate their regulatory role in TED.

The miRs are important regulators of gene expression. However, there are limited data on the involvement of miRs in the pathogenesis of TED. Up to date, only miR-130a, miR-146a and miR-155 and miR-1287-5p have been reported in TED [48–50]. In the present work, we predicted 4581 miRs targeting genes in the coexpression network involved in TED. The most prevalent miRs were hsa-miR-144, hsa-miR-3662, hsa-miR-12136 and hsa-miR-3646. Thus, our study provided a new set of miRs with relevant potential functions in the pathogenesis of TED. Further studies based on these miRs might enlighten our understanding on the pathogenesis of TED and open new avenues for the development of therapeutics for this disease.

To date, there is no effective drug for the treatment of TED. Few studies have indicated that some drugs including teprotumumab, tocilizumab, rituximab and mycophenolate can improve the outcomes of TED [51]. Herein, we predicted a set of drugs that could target the predicted gene coexpression network. Enzyme inhibitors and chlorodiphenyl (54% chlorine), finasteride, nefazodone, sodium arsenate, beta-methylcholine, propiconazole, pentachlorophenol, glucose, thimerosal, thapsigargin, succimer were the drugs with the highest number of target genes. The predicted genes may be efficient in the treatment of TED, which needs further experimental screening and validation.

Our study presents some limitations. Although TED-specific genes were found through the combined use of DEGs, lmQCM and WGCNA, due to the small sample size we used, there may be some deviations. In addition, our results were only based on public data, and more in vivo and in vitro experiments are still needed to verify our findings. We found 11 hub genes regulating the pathway of ubiquitin and proteasome through PPI analysis, but the role of these genes in the occurrence and development of TED still needs in-depth validation. In addition, we tried to identify the association of genes in the TED coexpression network to other diseases in ToppGene, but the analysis output revealed no disease association (no output data), which implied that the coexpression network might be specific to TED; however, tremendous work is needed to confirm this observation. We hereby call on more colleagues to experimentally verify our results.

## Conclusions

By harnessing different bioinformatics approaches, we constructed a robust gene coexpression network for TED 11 hub genes involved in this disease. Furthermore, we predicted the TFs, miRs and drugs that could target genes in the coexpression network. The functions of the coexpression network were also identified and indicated the important implication of protein ubiquitination in TED. The role of the identified hub genes in the formation and development of TED has not been reported so far. So, this is the first study to propose that these 11 hub genes may be related to the pathological process of TED, but more evidence needs to be given in future research. In the future, we will conduct more experimental research to explore the central genes of TED, so as to lay the foundation for diagnosing the occurrence and development of TED and developing new therapeutic targets for the treatment of TED.

## Methods

### Data collection and preprocessing

**Fig. 7:**
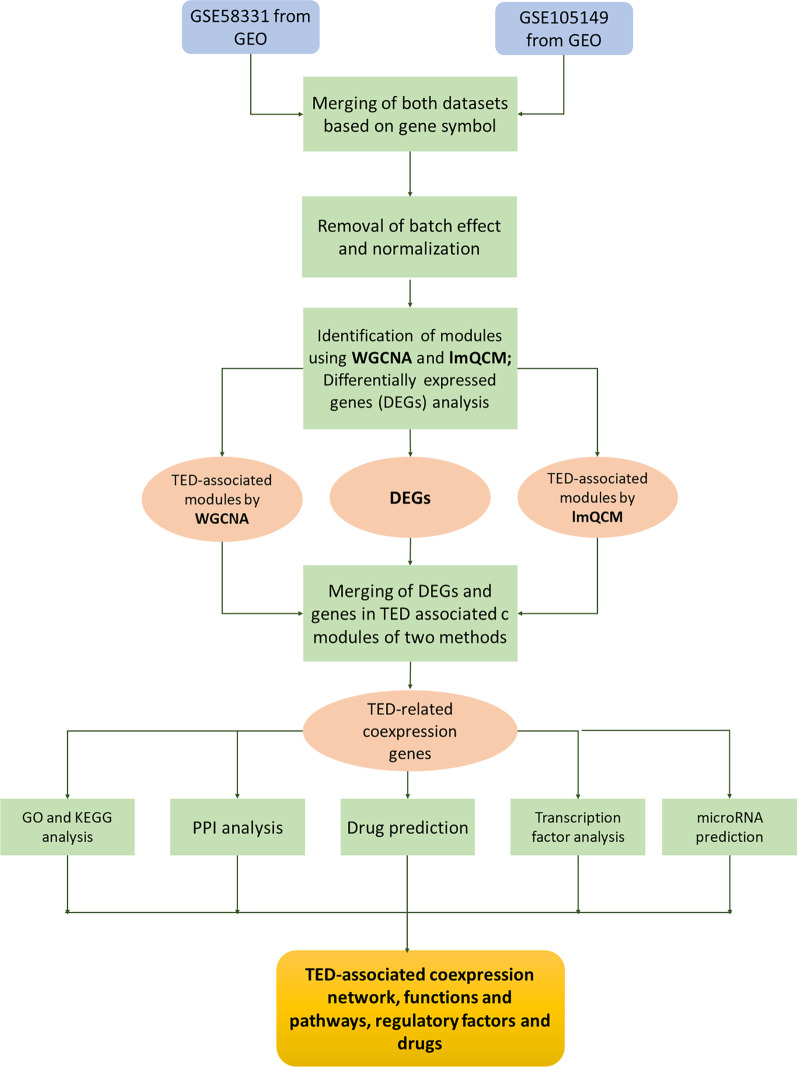
Workflow to identify TED-specific coexpression modules and TED-associated pathway and hub genes

The data used in this study were obtained from two datasets in the GEO database (https://www.ncbi.nlm.nih.gov/): GSE105149 (containing four TED samples and seven Normal samples) and GSE58331 (containing 35 TED samples and 29 Normal samples). The platform of the two datasets was GPL570. The array probes of each dataset were mapped to their respective gene IDs using corresponding array annotations. Probes matching multiple genes were represented by those with the highest average expression level. As shown in Fig. 7, the two datasets were merged into a matrix containing 22,880 genes and 75 samples. The batch effect of the merged matrix was removed by the R “sva” package (http://www.bioconductor.org/packages/release/bioc/html/sva.html) (Fig. 1A). The normalizeBetweenArrays function in the R “limma” package (http://www.bioconductor.org/packages/release/bioc/html/limma.html) was used to perform normalization of the data in the merged matrix (Fig. 1B).

### Gene coexpression network construction and modules selection

WGCNA network was performed to identify TED-related modules based on the preprocessed matrix. Firstly, the similarity of gene coexpression between gene *m* and gene *n* was defined as *S*_mn_ =|cor (*m*, *n*)|. Secondly, a power function was used to correlate adjacency between the genes: *a*_mn_ = power (*S*_mn_, *β*) =|*S*_mn_| ^*β*^. Thirdly, the gradient method (the power value ranging from 1 to 30) was used to test scale independence and mean connectivity. When the value was above 0.80 [11], a proper power value was screened out to establish a scale-free network. Finally, the adjacency matrix was converted into a topological overlap matrix and the module was screened out by using hierarchical average linkage clustering analysis, which was a gene dendrogram with a minimal size of 60. Then, 400 genes were randomly selected for heatmap drawing.

Additionally, we performed the local maximized quasi-clique merger (lmQCM) network mining [26] based on the preprocessed matrix. First, the samples in the TED matrix were divided into two groups: TED group and normal group. Then, the Pearson correlation coefficient (PCC) of each pair of genes in the TED and normal groups was calculated separately. Finally, we obtained coexpression networks associated with TED and normal groups. In the networks, nodes correspond to genes, and weight corresponds to PCC value. The lmQCM [26] was used to detect densely connected modules in the weighted network. The parameters for lmQCM were set as follows: gamma = 0.55, *t* = 1, lambda = 1, beta = 0.4, minimum cluster size = 10. The lmQCM is available both in web version (https://apps.medgen.iupui.edu/rsc/tsunami/) and R package in CRAN as “lmQCM.”

To identify the module associated with TED in weighted networks constructed by WGCNA and lmQCM, respectively, we did the following processing. Based on the modules identified in the previous step, we used the first principal component of each module (also named module eigengene, ME) to represent the expression levels of genes in each module. The correlation between MEs and TED was evaluated using PCC, and the modules with the highest or lowest PPC were selected as the TED-specific modules for the coexpression networks from WGCNA or lmQCM.

### Identification of DEGs

The differential expression analysis based on the merged matrix was performed by “limma” package in R with the threshold of |log2FC|> 0.263 and adj.* p* value < 0.05. The expression levels of the top 10 up-regulated and down-regulated genes (ranked by |log2FC|) were used to draw the heatmap by R “pheatmap” package. The volcano plot was visualized by the R “ggpubr” (https://cran.r-project.org/web/packages/ggpubr/index.html) and “ggthemes” (https://cran.r-project.org/web/packages/ggthemes/index.html) packages. The top 10 significant genes in up-regulated and down-regulated genes (ranked by adj.* p* value) were highlighted with gene symbols.

### Overlap of DEGs and genes in TED-specific modules

We merged the DEGs and genes in TED-specific modules and got TED-specific genes. The Venn diagram analysis was performed using the ggvenn library in R programming software to identify the intersection between lists of genes from DEGs, the blue module in WGCNA and the red module in lmQCM.

### ToppGene Suite analysis for prediction of TFs, microRNAs and candidate drugs

The prediction of TFs, microRNAs and candidate drugs based on the TED-specific genes were explored by analysis in ToppGene Suite (https://toppgene.cchmc.org/). The *p* value cutoff was set to 0.01.

### Protein–protein interaction (PPI) network construction

To explore the interaction between proteins coded by TED-specific genes, PPI analysis was performed. Herein, we uploaded the TED-specific genes to the Search Tool for the Retrieval of Interacting Genes/Proteins (STRING, https://string-db.org/cgi/), which is a database of known and predicted protein–protein interaction. We selected the organism “Homo sapiens” and downloaded *.TSV and *.png files from the STRING database. The network was also imported into Cytoscape 3.4.0 software for visualization. The MCODE plug-in in Cytoscape was used for the identification of gene clusters. The nodes with no interaction with other proteins in the PPI network were removed, and only genes with node degree higher than ten were visualized in Cytoscape.

### Functional enrichment analysis

The biological function of the genes was investigated by the *R* “clusterProfiler” package [52]. The GO terms and pathways were sorted by adjusted *p* values (adj. *p* value), and adj. *p* values < 0.05 were considered significant. The top 10 terms of GO biological process, cellular component and molecular function and the top 10 pathways of the KEGG pathway were selected and visualized by the *R* “ggplot2” package [53].

## Supplementary Information

**Additional file 1: Table S1** Results of the functional enrichment analysis of genes in differentially expressed genes (DEGs) as the output of cluster profiler analysis.

**Additional file 2: Table S2** Results of the functional enrichment analysis of genes in the blue module identified in WGCNA analysis as the output of cluster profiler analysis.

**Additional file 3: Table S3** Results of the functional enrichment analysis of genes in the red module identified in lmQCM analysis as the output of cluster profiler analysis.

**Additional file 4: Table S4** Results of the functional enrichment analysis of the 497 TED-associated coexpression genes as the output of cluster profiler analysis.

**Additional file 5: Table S5** Detailed information of predicted transcription factors.

## Data Availability

All data generated or analyzed during this study are included in this published article.

## Abbreviations

TED: Thyroid eye disease
TAO: Thyroid-associated ophthalmopathy
WGCNA: Weighted gene coexpression network analysis
TF: Transcription factor
TOM: Topological overlap matrix
